# Fatigue Life Analysis of Titanium Torsion Spring Based on Continuous Damage Mechanics

**DOI:** 10.3390/ma18020221

**Published:** 2025-01-07

**Authors:** Dehai Meng, Changming Zhang, Fan Yang, Feixiang Duan

**Affiliations:** 1 School of Mechanical Engineering, Shaanxi University of Technology, Hanzhong 723001, China; mdh2829878219@163.com (D.M.); yang_fan@snut.edu.cn (F.Y.); 18792724654@163.com (F.D.); 2 Shaanxi University Enterprise Joint Research Center for Advanced Manufacturing of Aircraft Landing Gear and Performance Testing of Aviation Components, Shaanxi University of Technology, Hanzhong 723001, China; 3 Engineering Research Center of Manufacturing and Testing for Landing Gear and Aircraft Structural Parts, Universities of Shaanxi Province, Hanzhong 723001, China

**Keywords:** fatigue damage, CDM, torsion springs, Ti-6Al-4V, finite element methods

## Abstract

In this study, a titanium alloy torsional spring used in aviation was taken as the research subject. Aiming at the fatigue life prediction problem of this spring, the life analysis of the titanium alloy torsional spring was performed using a customized UMAT subroutine based on the theory of continuous damage mechanics. Several sets of life prediction models and tests were compared. The fatigue lives of the springs at 60, 80, 100, and 120 degrees were 45,070, 65,067, 99,677, and 181,322 cycles, respectively. Compared with other fatigue life prediction methods, the fatigue life calculated by the customized subroutine was the most consistent with the fatigue life of the titanium alloy torsion spring tests. The average relative error between the measured experimental life value and the predicted value was 2.04%, which is less than 5%, meeting engineering measurement requirements. The effectiveness and applicability of the proposed model and method were verified, and the time and economic cost caused by excessively long experimental cycles were reduced. This helps improve the accuracy of fatigue life prediction for this titanium alloy torsional spring and provides analysis support for subsequent structural optimization and improvement.

## 1. Introduction

Every year, safety accidents caused by the failure of mechanical parts not only cause huge economic losses but also pose a great threat to the life safety of personnel, which has a more obvious impact in the aviation field [1,2]. In a study on the failure causes of aircraft components, the failure forms and frequencies of aircraft components were statistically analyzed, and it was found that the incidence of fatigue failure was 55% [3], and fatigue failure accounted for a very high proportion among all failure forms. The normal service of a titanium alloy torsion spring, a key part of the landing gear ejection system of an aviation aircraft, is very important for the operation and flight safety of the aircraft and has a significant impact on the safety and reliability of the mechanical system. The fracture of the spring will lead to the abnormal operation of the mechanical system reset function, and it is necessary to know the cycle life of the spring under different working load conditions. In addition to working load and external environmental conditions, metal materials are also an important factor affecting the fatigue performance of mechanical components [4]. Common metal materials, such as aluminum alloys, high-strength steel and nickel-based high-temperature alloys, cannot meet the requirements of manufacturing aircraft parts in terms of quality, strength, corrosion resistance, and high-temperature stability, while titanium alloys not only display excellent performance in these aspects but also have high strength, low density, and good corrosion resistance, which have been widely used in mechanical and aerospace engineering [5]. At present, the titanium alloy widely used at home and abroad is Ti-6Al-4V, which accounts for about 65% of the entire titanium alloy in engineering applications [6]; therefore, Ti-6Al-4V became the material of choice for manufacturing this torsion spring.

In recent years, in the field of spring fatigue analysis and life prediction, people have made a series of explorations of spring fatigue life by various numerical simulations and experimental detection. Zhang [7] observed the fatigue fracture morphology of double torsion springs made of SUS 631 stainless steel; analyzed the origin of fatigue cracks and the influence of material defects on fatigue performance; performed finite element simulations using ANSYS Workbench; compared these with experiments; found that the area where the equivalent stresses were concentrated was the most likely location of damage; and proposed to improve the fatigue performance by mechanical surface treatment, such as shot peening, low plasticity burnishing, and laser impact machining, to improve the fatigue performance. Vizcaya [8] conducted a failure analysis of helical compression springs and found that the Wang–Brown criterion overestimated the fatigue life of springs, while the prediction results obtained by the Coffin–Manson model were relatively conservative, and the critical plane method could better predict the fatigue life of such springs. Pastorcic [9] established a helical spring finite element model applied to an automobile suspension system, carried out stress analysis under random variable dynamic load, and used the Matlab numerical program to conduct a minor cumulative damage assessment according to the Goodman failure criteria. Finally, the fatigue life prediction results of the spring have been given. The research results can provide an effective reference for further optimization design of automotive helical springs. Kong [10] adopted the conservative Morrow and Smith Watson topper (SWT) average stress correction method to simulate the fatigue life of plate springs and studied the fatigue life of automotive parabolic plate springs under multiple cycle loads under different road conditions. The influence of three different loads on the life damage value of the parabolic plate spring was obtained, and the fatigue failure time of the plate spring under different road conditions was estimated.

The previous studies on fatigue life were conducted on the fatigue life analysis of springs using different fatigue analysis models and experiments, but unfortunately, the fatigue life prediction of titanium torsion springs based on continuous damage mechanics is relatively rare and still needs to be applied and improved. The continuous fatigue damage model can effectively capture the damage accumulation during the fatigue process. This model usually considers the microstructure changes of materials, thus describing the damage evolution more truly [11,12,13], and has a good ability to predict the behavior of metals under complex loading conditions. Among the various prediction models, one of the most renowned ones was proposed by Chaboche et al. [14]. This model takes a phenomenological approach to evaluate the nonlinear progression of fatigue damage, considering it as a function dependent on both the maximum stress and average stress. Following this, Chaudonneret [15] expanded upon this model to make it suitable for multi-axis loading conditions. Additionally, Shang, Dattoma [16,17] also developed a nonlinear fatigue damage evolution model that incorporates the influence of variable loads. Lemaitre [18] developed a fatigue model that incorporates both micro-plasticity and micro-damage, with the aim to analyze the impact of cyclic external loads on material deterioration. This model introduces a damage variable D to describe the gradual accumulation of damage over time. By incorporating stress or strain into the damage field through an evolution equation, this approach provides a clear physical interpretation of the process of material degradation [19]. Based on the continuous damage mechanics theory, Zixu Guo [19] combined the damage variable with the multi-linear elastoplastic constitutive model, applied the low-cycle fatigue life prediction of turbine blades, and conducted experimental verification on full-size turbine blades. The prediction effect of the model was confirmed, and it is easy to extend the multi-axis loading condition. Xia, Q [20] proposed a method that combines the continuum damage mechanics–finite element method (CDM-FEM) with particle swarm optimization (PSO)-based techniques for predicting the low- and medium-circumferential fatigue life of perforated structures. Liu [21] suggested a fatigue damage model that integrates the critical plane approach, specifically designed to accommodate both proportional and non-proportional multi-axial loading conditions. Although these methods were applied to metals, such as titanium alloys, how to apply this theory to predict torsional springs in titanium alloys and design experiments to validate their predicted performance is an issue that needs to be investigated. Therefore, based on the previous research and experimental summary, the continuous damage mechanics are realized and applied to the life prediction and fatigue damage analysis of this aviation torsion spring by a numerical method.

A method for predicting the life of titanium alloy torsional springs based on continuous damage mechanics is introduced in this paper. The numerical implementation of the model is developed using ABAQUS software. The results show good accuracy compared with different multi-axis prediction models and experiments. The rest of this paper is organized as follows. The detailed mathematical model, the determination of its model parameters, and the implementation of the model algorithm are introduced in Section 2. Section 3 contains a description of the finite element model of the torsion spring and the fatigue experimental process, followed by a discussion of the predictive effect of the fatigue damage model in Section 4. Finally, the conclusion will be given in Section 5.

## 2. Finite Element Analysis and Experimental Method of Torsion Spring

### 2.1. Establishment of Torsion Spring Finite Element

**Figure 1:**
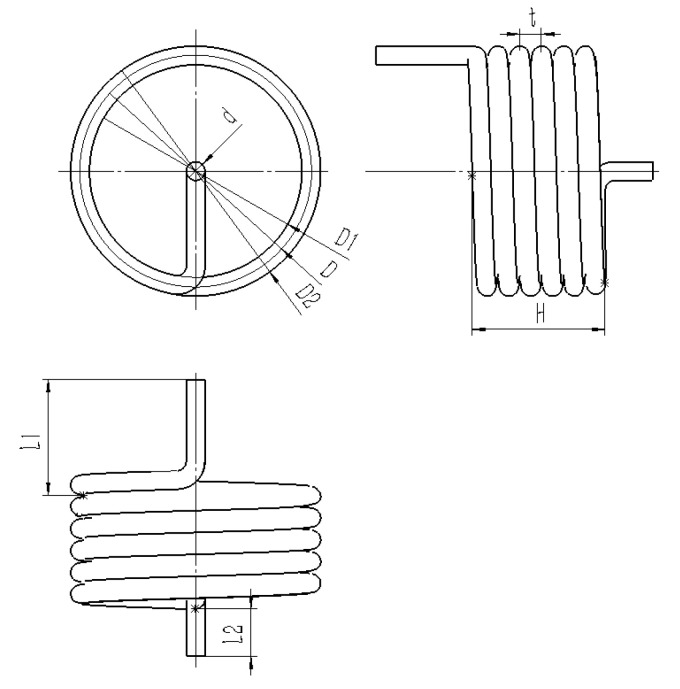
Torsion spring structure diagram.

The specific physical parameters of the torsion spring structure are as follows: diameter: d = 3 mm, middle diameter: D = 37 mm, inner diameter: D1 = 34 mm, outer diameter: D2 = 40 mm, pitch: t = 3.5 mm, torsion arm: L1 = 16 mm, torsion arm: L2 = 6 mm, effective length: H = 20.5 mm, and rotation direction: right. The structural parameters of the torsion spring are shown in Figure 1.

**Figure 2:**
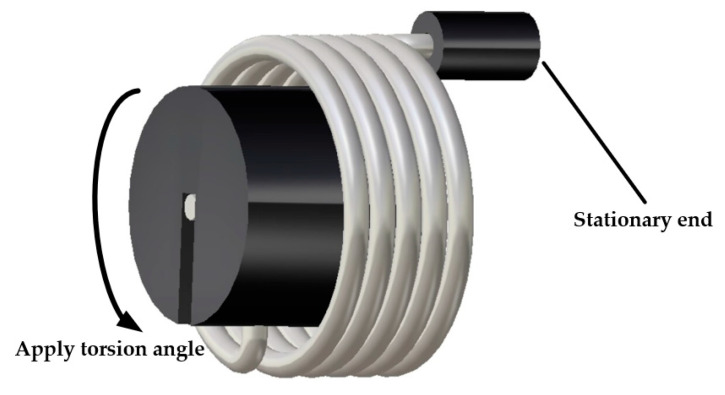
Schematic diagram of load and boundary conditions.

Finite element working condition settings: as shown in Figure 2, one end of the torsion arm of the torsion spring was fixed, and a load was applied to the other end. The spring was twisted from the initial position to the maximum torsion angle and then back to the initial position again in 1 s for one loading cycle. The ABAQUS/Standard solver was selected, and the length of the analysis step was 1, using automatic incremental steps. Since it was necessary to use a subroutine file for the simulation, the cell type of the mesh was in the form of a reduced integral and the hourglass control was changed to augmented.

The finite element model was established on the ABAQUS platform and numerically calculated. The C3D8 eight-node linear hexahedron element was used to mesh the model. To ensure the accuracy of the calculation results, a mesh sensitivity analysis was carried out on the model, and the stress of the torsion spring when twisted at 120 degrees under different mesh encryption conditions was calculated. As shown in Table 1, the resulting converged solution was obtained independent of the size of the finite element mesh.

**Figure 3:**
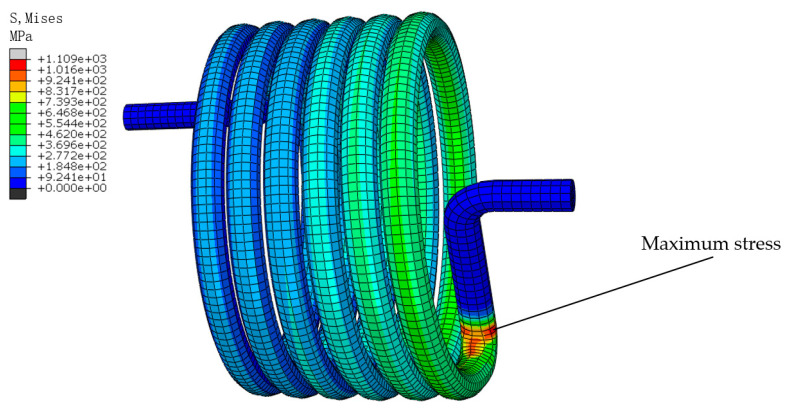
Maximum stress of torsion spring.

Finally, in order to ensure the computational efficiency and to take into account the stress convergence, this study selected a 0.5 mm mesh to mesh the model, where the finite element model contained 17,829 elements. Figure 3 shows the cloud diagram of the stress distribution when the torsion angle was 120 degrees. The cloud diagram shows that the maximum stress was located at the inflection point of the torsion arm of the spring, where $\sigma_{max}$ = 1109 MPa.

### 2.2. Fatigue Test Method of Titanium Alloy Torsion Spring

**Figure 4:**
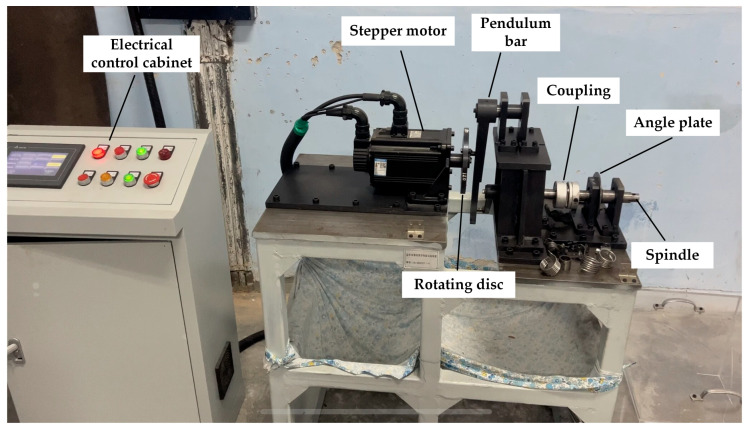
Fatigue strength test bench of titanium alloy torsion spring.

To measure the fatigue life of the torsion spring under real working conditions, the fatigue life test platform was used to evaluate the spring’s life at a temperature of 22 °C under room conditions. The fatigue life test platform of the torsion spring was driven by a stepping motor to rotate the porous disc, which was connected to a rocker through the slider, which enabled the rocker to swing within a specific range of angles. The rocker’s arm was connected to the rotating shaft of the table via a coupling, which caused the shaft to rotate at a specific angle. One end of the torsion spring was fixed to the bearing seat, while the other end was connected to the rotating shaft of the table. A pointer was attached to the rotating shaft of the table to indicate the current rotation angle. When the motor worked, the rocker swung, and the rotating shaft of the table rotated within the set angle range. It drove the torsion spring to rotate. The loading rotation rate of the spring on the experimental bench was 2π/3 rad/s, and the torsion angles of the spring were 60 degrees, 80 degrees, 100 degrees, and 120 degrees. The torsional spring fatigue life test bench, shown in Figure 4, mainly included the stepper motor, pendulum bar, coupling, angle plate, spindle, and rotating disc.

**Figure 5:**
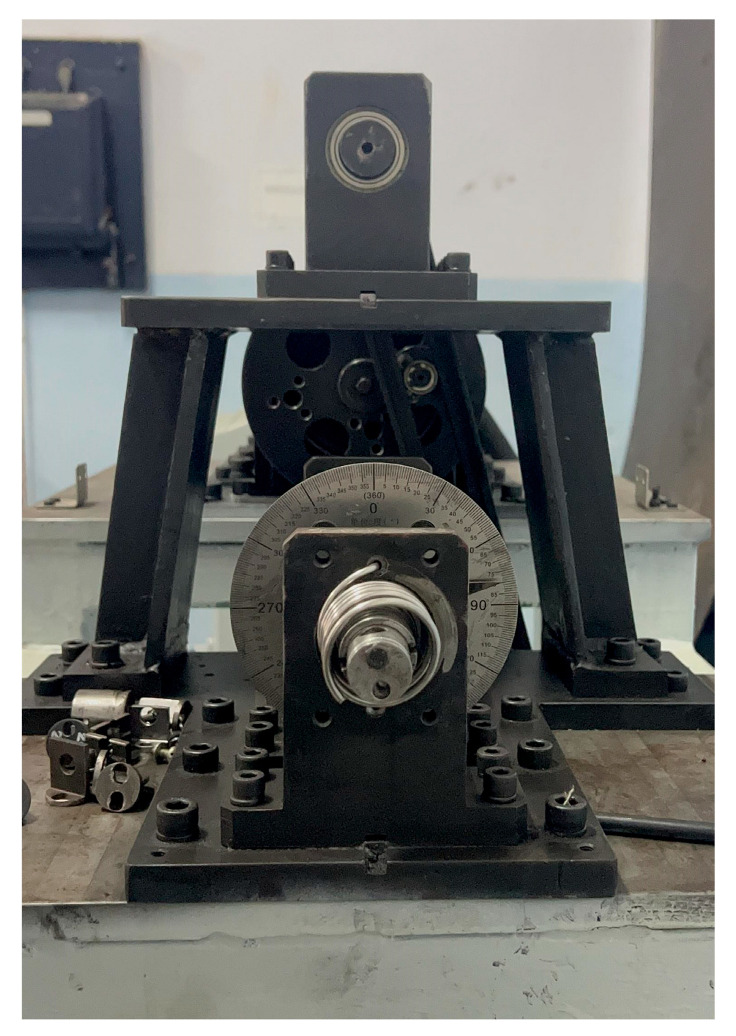
Spring break test stand stopped rotating.

The working angle of the titanium torsion spring was 120 degrees. To verify the accuracy of the fatigue prediction based on the continuous damage mechanics model in all aspects, the torsion spring fatigue test rig was selected to work without an initial pre-torsion angle, and then twisted to 60 degrees, 80 degrees, 100 degrees, and 120 degrees, as shown in Figure 5. When the spring broke, the test rig stopped working, and the resulting fatigue life of the spring results are shown in Table 2.

## 3. Materials and Methods

### 3.1. Damage-Coupled Chaboche Plastic Constitutive Model

**Figure 6:**
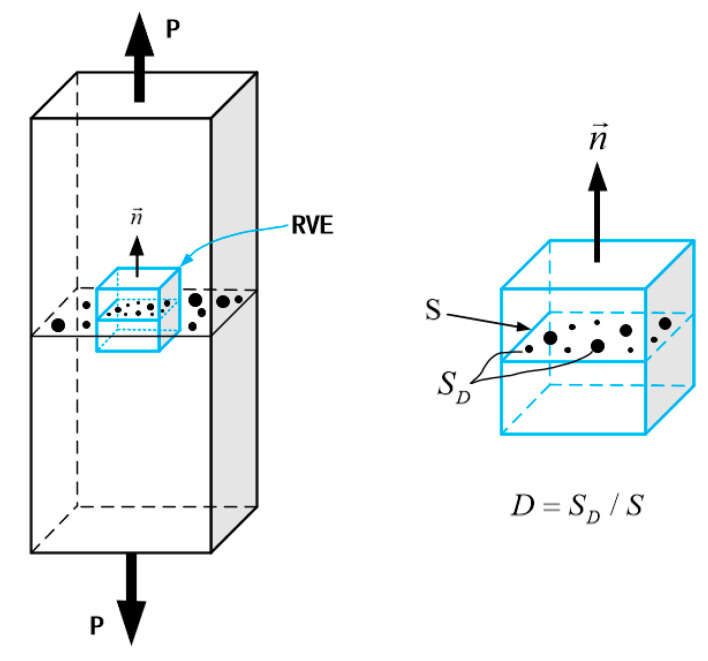
Representative volume element.

In the field of engineering, continuum mechanics can be described by representative volume units (RVEs), where all mechanical properties are represented by homogenized variables, as shown in Figure 6 [22]. It should be emphasized that the RVE is the smallest material unit in the continuum mechanics calculation or analysis, and its size is determined by the microstructure of the material. For example, for metallic materials, the commonly used analytical size is about 0.1 mm³. Under the framework of continuous damage mechanics, the damage variable *D* is introduced to characterize the deterioration of the material, assuming that the damage of the material is uniformly distributed inside the RVE. The damage variable *D* is related to the direction of the normal vector n and can be defined by the reduction in the effective bearing area of the RVE, specifically as follows:

(1) $$D=\frac{S_{D}}{S}=\frac{S-\tilde{S}}{S}$$

In this formula, $\tilde{S}$ represents the effective bearing area and S represents the total area of the section representing the volume unit (RVE). When $\tilde{S}=S$, that is, D=0, the material is not damaged; when $\tilde{S}=0$, that is, D=1, it means that the material is destroyed, and it is considered that the fatigue crack has been generated. Based on the concept of effective stress and the assumption of strain equivalence [23], the definition of the damage variable is expressed through the reduction of the elastic modulus, and the relationship between the damage variable and the equivalent elastic modulus of the representative volume unit (RVE) is established, namely,

(2) $$D=1-E/E_{0}$$

where E is the equivalent modulus of elasticity of the damaged RVE and $E_{0}$ is the initial modulus of elasticity of the material. Considering small deformations, the total strain $\varepsilon_{ij}$ can be expressed as

(3) $$\varepsilon_{ij}=\varepsilon_{ij}^{e}+\varepsilon_{ij}^{p}$$

where $\varepsilon_{ij}^{e}$ and $\varepsilon_{ij}^{p}$ are the elastic strain and plastic strain, respectively. According to the principle of strain equivalence [23], the form of elastic strain is as follows:

(4) $$\varepsilon_{ij}^{e}=\frac{1+\nu }{E_{0}}\frac{\sigma_{ij}}{1-D}-\frac{\nu }{E_{0}}\frac{\sigma_{kk}}{1-D}\delta_{ij}$$

where ν is Poisson’s ratio, $\sigma_{ij}$ represents Cauchy’s stress, and $\delta_{ij}$ is the Kronecker delta symbol. The von Mises yield function with damage can be expressed as follows:

(5) $$f=\sqrt{\frac{3}{2}(\frac{s_{ij}}{1-D}-\alpha_{ij})(\frac{s_{ij}}{1-D}-\alpha_{ij})}-Q$$

where $s_{ij}$ is the deviatoric portion of the stress and $\alpha_{ij}$ is the deviatoric portion of the back stress. Q is the yield surface radius, which is defined as

(6) $$\dot{Q}=\dot{\lambda }b(Q_{\infty }-Q)$$

The plastic multiplier, denoted as $\dot{\lambda }$, is determined by the condition of plastic flow consistency, where $\dot{f}=f=0$. The motion-hardening behavior is represented using the nonlinear motion-hardening model (NLKH) proposed by Chaboche [24], with b and $Q_{\infty }$ being material constants.

(7) $$\alpha_{ij}=\sum_{k=1}^{M}\alpha_{ij}^{\left(k\right)}$$

The evolution process of the plastic strain component [23] can be obtained:

(8) $${\dot{\varepsilon }}_{ij}^{p}=\frac{3}{2}\frac{\dot{\lambda }}{1-D}(\frac{s_{ij}}{1-D}-\alpha_{ij})/{\left(\frac{s_{ij}}{1-D}-\alpha_{ij}\right)}_{eq\nu }$$

(9) $$\dot{p}=\sqrt{\frac{2}{3}{\dot{\varepsilon }}_{ij}^{p}{\dot{\varepsilon }}_{ij}^{p}}=\frac{\dot{\lambda }}{1-D}$$

(10) $${\dot{\alpha }}_{ij}^{\left(k\right)}=\left(\frac{2}{3}C_{k}{\dot{\varepsilon }}_{ij}^{p}-\gamma_{k}\alpha_{ij}^{\left(k\right)}\dot{p}\right)(1-D)$$

Here, $\dot{p}$ represents the cumulative plastic strain rate. In Equation (9), $C_{k}$ and $\gamma_{k}$ are material constants that are determined through experimental testing.

### 3.2. Damage Evolution Model

According to the stress-controlled low-cycle fatigue test conducted by Kang [25], it can be considered that damage D is divided into elastic damage and plastic damage, which depend on the cyclic stress and cumulative plastic strain, respectively. As a result,

(11) $$D=D_{e}+D_{p}$$

#### 3.2.1. Plastic Damage Model

The evolution rule for plastic damage $D_{p}$, taking into account the stress triaxiality effect [26], is given by

(12) $${\dot{D}}_{p}={\left(\frac{\sigma_{eqv}^{2}R_{v}}{2E_{0}S{\left(1-D_{p}\right)}^{2}}\right)}^{m}\dot{p}$$

(13) $$R_{v}=\frac{2}{3}(1+v)+3(1-2v){\left(\sigma_{H}/\sigma_{eqv}\right)}^{2}\;\mathrm{if}\;\sigma_{H}\geq 0$$

In these formulas, S and *m* represent the characteristic parameters of the material; $R_{v}$ represents the stress triaxial function; while $\sigma_{H}$ and $\sigma_{eqv}$ correspond to the hydrostatic stress and von Mises stress, respectively. The existing literature has a limited understanding of the damage mechanism under compressive stress, and its specific manifestation is still unclear. To effectively extend the plastic damage model to compressive stress states, this study employed a conservative approach. The stress triaxiality function under compressive stress conditions is represented as follows:

(14) $$R_{v}=\frac{2}{3}\left(1+v\right)\;\mathrm{if}\;\sigma_{H}<0$$

#### 3.2.2. Elastic Damage Model

The thermodynamic formula of elastic damage has been described in detail in reference [23]. In the scenario of cyclic loading with multiple axes, the damage rate equation for a nonlinear continuous damage model (NLCD) can be formulated as

(15) $${\dot{D}}_{e}=\frac{dD}{dN}={\left[1-{\left(1-D\right)}^{\beta +1}\right]}^{\alpha }\cdot {\left[\frac{A_{II}}{M_{0}(1-3b_{2}\sigma_{H,mean})(1-D)}\right]}^{\beta }$$

Here, N denotes the number of cycles until failure, while β, $M_{0}$, and $b_{2}$ are the material constants identified through fatigue testing. $A_{II}$ is the octahedral shear stress amplitude, expressed as

(16) $$A_{II}=\frac{1}{2}{\left[\frac{3}{2}(S_{ij,max}-S_{ij,min})\cdot (S_{ij,max}-S_{ij,min})\right]}^{1/2}$$

In one loading cycle, the deviatoric stress tensor’s ij component has a maximum value of $S_{ij,max}$ and a minimum value of $S_{ij,min}$. The average hydrostatic stress, denoted as $\sigma_{H,mean}$ in Equation (14), is defined as follows:

(17) $$\sigma_{H,mean}=\frac{1}{2}(\sigma_{H,max}+\sigma_{H,min})$$

where $\sigma_{H,\max}$ and $\sigma_{H,\min}$ are the maximum and minimum hydrostatic stresses of a loading period, respectively. Also, in Equation (14), the parameter α is given by the formula

(18) $$\alpha =1-a\langle \frac{A_{II}-A_{II}^{*}}{\sigma_{u}-\sigma_{eqv,\max}}\rangle$$

In a loading period, the maximum von Mises stress is denoted as $\sigma_{eqv,\max}$. The Sines fatigue limit in this model can be approximately expressed as

(19) $$A_{II}^{*}=\sigma_{l0}(1-3b_{1}\sigma_{H,mean})$$

$\sigma_{l0}$ represents the fatigue limit at zero mean stress, while $b_{1}$ denotes the material constant. The cycle count until failure is determined by integrating Equation (14) from D = 0 to D = 1:

(20) $$N_{F}=\frac{1}{1+\beta }\frac{1}{aM_{0}^{-\beta }}\frac{\langle \sigma_{u}-\sigma_{max}\rangle }{\langle \sigma_{a}-\sigma_{l0}(1-b_{1}\bar{\sigma })\rangle }{\left[\frac{\sigma_{a}}{1-b_{2}\bar{\sigma }}\right]}^{-\beta }$$

### 3.3. Determination of Material Parameters and UMAT Calculation Method

#### 3.3.1. Parameters of the Elastoplastic Constitutive Model

The determination of the back stress constant is based on experimental data from stress–strain curves of uniaxial monotonic stretching [27]. In this study, it was assumed that isotropic hardening was negligible, i.e., the yield surface dimensions were kept constant. In the finite element realization, the parameters b and $Q_{\infty }$ were set to 1.0 and 0. At this point, the isotropic hardening transforms to

(21) $$Q=\sigma_{y}$$

where $\sigma_{y}$ is the initial yield stress.

In the scenario of uniaxial loading, the exponential saturation equation is utilized to approximate each component of back stress:

(22) $$\alpha^{\left(k\right)}=\frac{C_{k}}{\gamma_{k}}(1-e^{-\gamma_{k}\varepsilon_{p}})$$

Then, the stress–strain curve is expressed as

(23) $$\sigma =\sigma_{y}+\sum_{k=1}^{M}\frac{C_{k}}{\gamma_{k}}(1-e^{-\gamma_{k}\varepsilon_{p}})$$

where σ and $\varepsilon_{p}$ are the stress and plastic strain, respectively.

**Figure 7:**
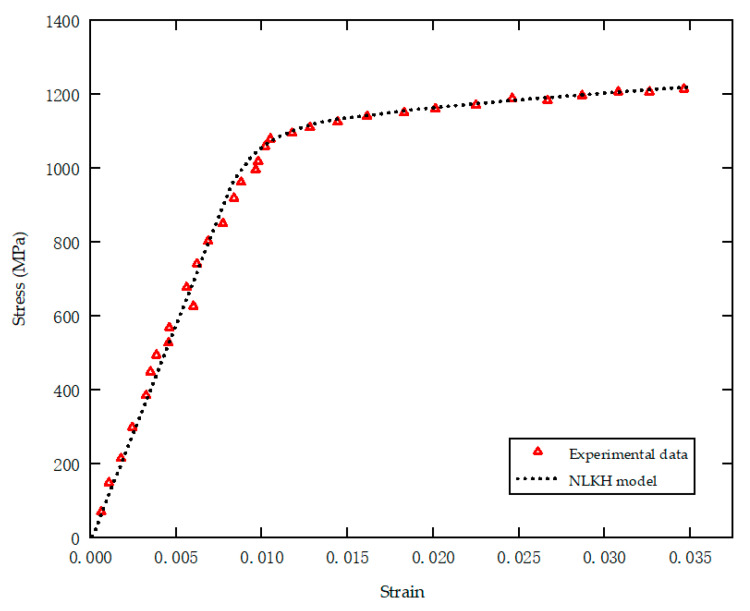
Uniaxial monotonic tension stress–strain curve for Ti-6Al-4V.

According to the experimental data, the least squares method was used to determine the parameters, and the data-fitting results are shown in Figure 7. Table 3 provides a comprehensive overview of the mechanical characteristics and material attributes associated with Ti-6Al-4V.

#### 3.3.2. Parameter Identification of Damage Evolution Model

In the field of engineering, continuum mechanics can be described using representative volume elements for the two different damage evolution models, the elastic and plastic damage models, where several parameters need to be determined. Fatigue tests on standard specimens were used to obtain the parameters in the damage evolution models.

##### Parameter Identification of Elastic Damage Evolution Model

In Equation (20), $\sigma_{max}$ represents the maximum stress, while $\sigma_{a}$ denotes the stress amplitude and $\bar{\sigma }$ signifies the average stress.

The ultimate tensile stress $\sigma_{u}$ is derived from the stress–strain curve of unidirectional tension. Factors $\sigma_{l0}$ and $b_{1}$ are established using fatigue limit data at various mean stresses.

**Figure 8:**
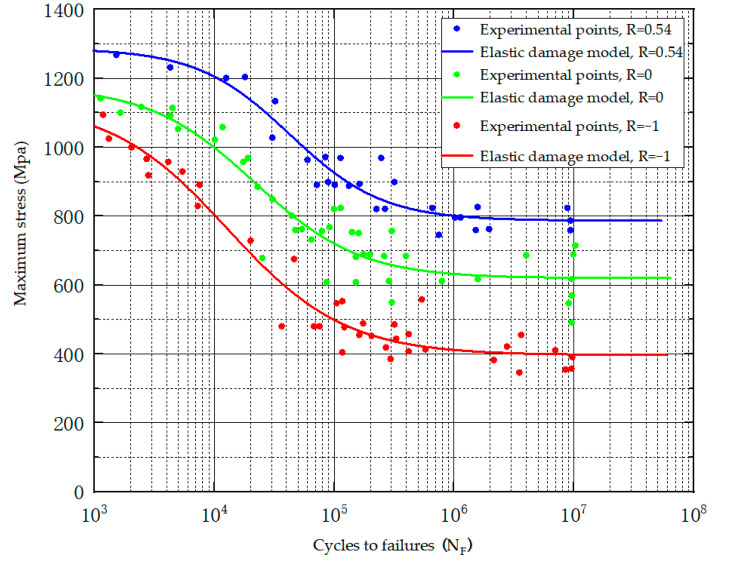
Test fatigue data and prediction curves of standard specimens.

The parameters β and $aM_{0}^{-\beta }$ are derived from stress control fatigue tests under full reverse-loading conditions. The parameters are obtained by fatigue tests under different mean stress conditions. The method proposed by Zhang [28] was adopted to obtain the parameter a. The fatigue test data for the Ti-6Al-4V material were sourced from the literature [27], and the associated parameters of the elastic damage evolution model are presented in Table 4. Figure 8 illustrates the test fatigue data, along with the predicted curve for the standard sample.

##### Parameter Identification of Plastic Damage Evolution Model

The integral equation of the damage evolution model and the plastic strain curve concerning the number of cycles were utilized to derive the parameters for the damage evolution model. By employing the Coffin–Manson law, we could express the relationship between plastic strain and cycle number as an equation:

(24) $$\frac{\Delta \varepsilon_{p}}{2}=\varepsilon_{f}{\left(2N_{f}\right)}^{c}$$

where $\varepsilon_{f}$ is the fatigue ductility coefficient and c is the fatigue ductility index. The comprehensive damage evolution equation, Equation (15), under uniaxial conditions is

(25) $$N_{F}=\frac{1}{2(2m+1)\Delta \varepsilon_{p}}{\left(\frac{2ES}{{\left(\sigma_{max}\right)}^{2}}\right)}^{m}$$

A cyclic stress–strain curve was adopted:

(26) $$\sigma_{max}=K^{\prime }{\left(\frac{\Delta \varepsilon_{p}}{2}\right)}^{n^{\prime }}$$

As the parameters obtained from the experiment, $K^{\prime }$ and *n*′ were utilized. Consequently, Equation (25) can be expressed as

(27) $$N_{F}=\frac{1}{2(2m+1)}{\left(\frac{2^{1+2n^{\prime }}ES}{{\left(K^{\prime }\right)}^{2}}\right)}^{m}{\left(\Delta \varepsilon_{p}\right)}^{-(1+2mn^{\prime })}$$

By utilizing the data from the plastic strain versus cycle number curve [29], the values of $\varepsilon_{f}$, c, $K^{\prime }$, and $n^{\prime }$ could be extracted. Subsequently, the parameters for the plastic damage evolution model were established and are presented in Table 5.

### 3.4. UMAT Subroutine Calculation Method

**Figure 9:**
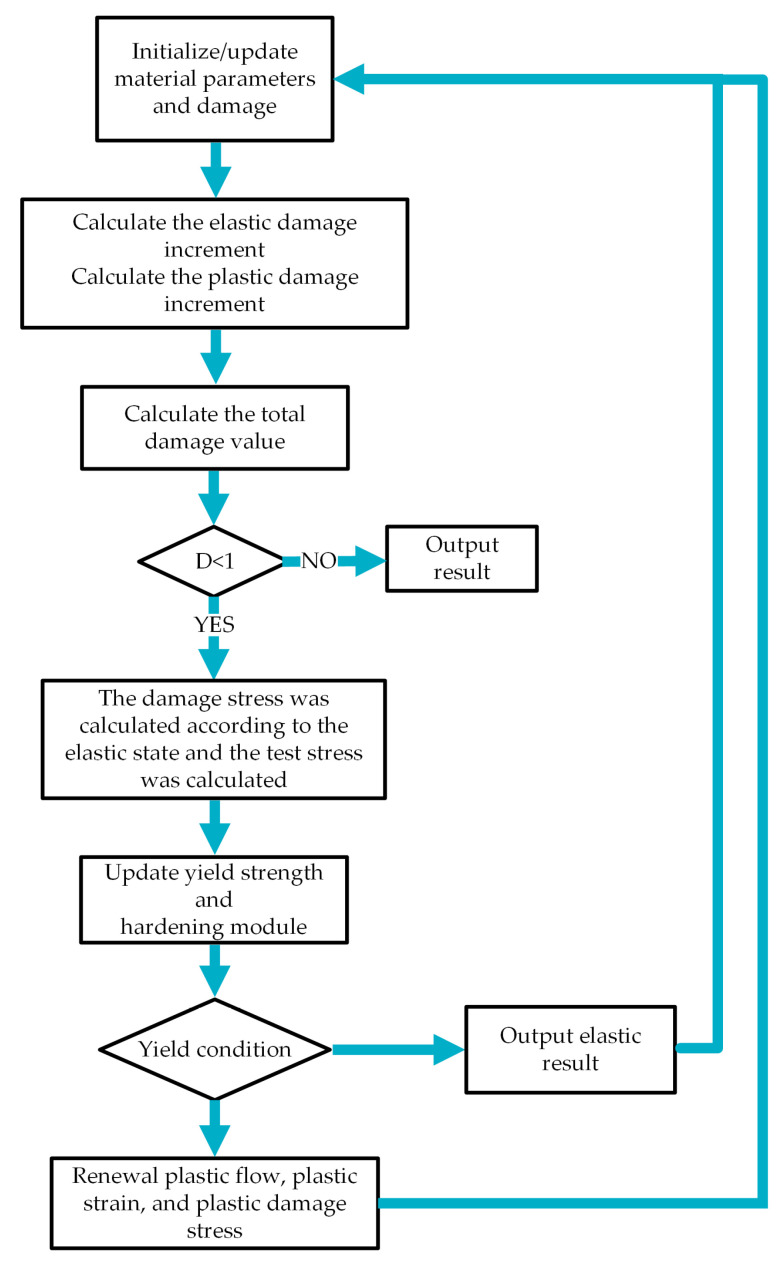
Simplified UMAT subroutine block diagram.

The Chaboche constitutive model for damage coupling and the damage evolution equation was integrated using a user material subroutine. To apply the damage-coupled elastoplastic constitutive model and damage evolution model to all material integral points, a custom subroutine was invoked at the start of each time increment. At the end of each time increment, updates were made to the stress and associated state variables, followed by updating the corresponding Jacobian matrix [30,31,32,33,34]. To reduce the computational costs associated with simulating each loading cycle, a jump cycle approach was employed in the numerical implementation. This assumes that the stress, cumulative plastic strain, and damage variables remain constant over a finite period. The simplified flow chart for this numerical algorithm can be seen in Figure 9, while further details on the calculation process can be found in references [35,36,37].

(1) Material parameters were set to their initial values, with an initial damage value of zero. The stress history at each integration point subjected to cyclic loading was obtained by solving the elastic damage constitutive equation, Equation (4), utilizing the ABAQUS solver.

(2) The increment and cumulative value of elastic damage were determined by solving Equation (15). Due to the large fatigue life value, it is time-consuming to calculate all the cycle processes individually. To address this, we adopted the jump algorithm [38], assuming that the damage value and stress–strain field remained constant during ΔN cycles. To ensure accuracy and save computational costs, we set ΔN as 1% of the journal fatigue prevention life. We calculated the elastic damage evolution rate and updated the accumulation value accordingly. The cumulative period $N_{\left(i+1\right)}=N_{\left(i\right)}+\Delta N$ was also updated.

(3) The increment of plastic damage and its cumulative value were calculated by solving Equation (12). The rate of plastic damage progression was determined and the accumulated elastic damage was updated accordingly.

(4) The cumulative value of the total damage was calculated by combining the increments of elastic and plastic damage. The damage level was assessed at each integral point. If the damage level reached 1, the program was terminated and the fatigue life was displayed. Otherwise, the method proceeded to step (6).

(5) The solution for Equation (4) was obtained, which resulted in the update of the stress based on the elastic condition. Subsequently, the test stress was determined using the von Mises yield criterion. The revised yield strength was calculated by considering the equivalent plastic strain from the previous cycle.

(6) The yield was evaluated to establish whether the requirements were satisfied. In case the yield condition was not achieved, the elastic outcome from step (5) was output as the result and substituted for the subsequent cycle. If the yield condition was attained, the method proceeded to step (7).

(7) The plastic flow was revised by applying the Newton method to compute the equivalent plastic strain, followed by an update of the stress damage. The outcome was then returned for use in the next iteration.

## 4. Analysis of Fatigue Life Results

### 4.1. Fatigue Fracture Prediction

**Figure 10:**
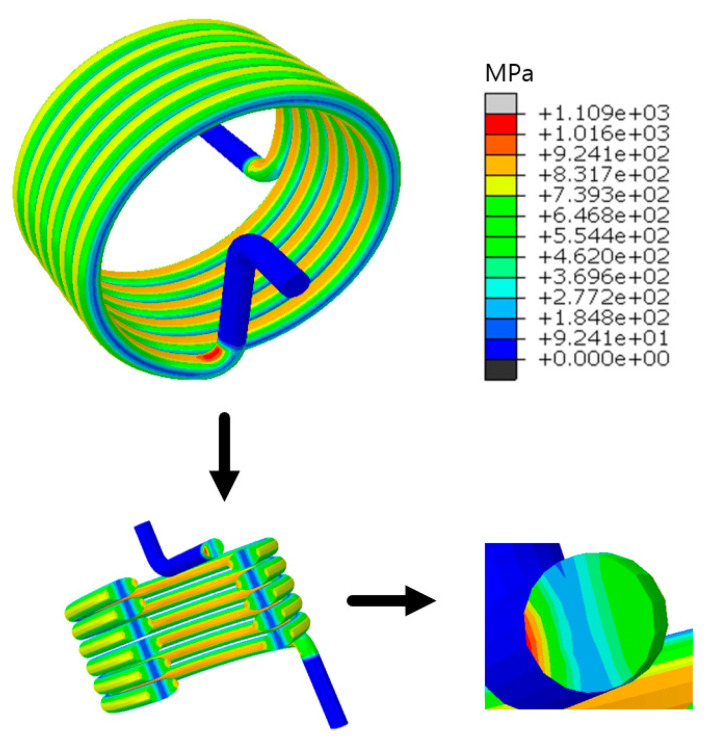
Stress distribution of the section at the maximum stress.

The model result file, calculated by the continuous damage force subroutine, was imported into ABAQUS to view the cloud image results of the titanium alloy torsion spring. The stress distribution at the cross-section of the spring could be seen by extracting the stress at the cross-section of the maximum stress. As shown in Figure 10, the stress on the innermost part of the spring was greater than that on the outermost part, and the stress in the middle area of the spring was the least.

**Figure 11:**
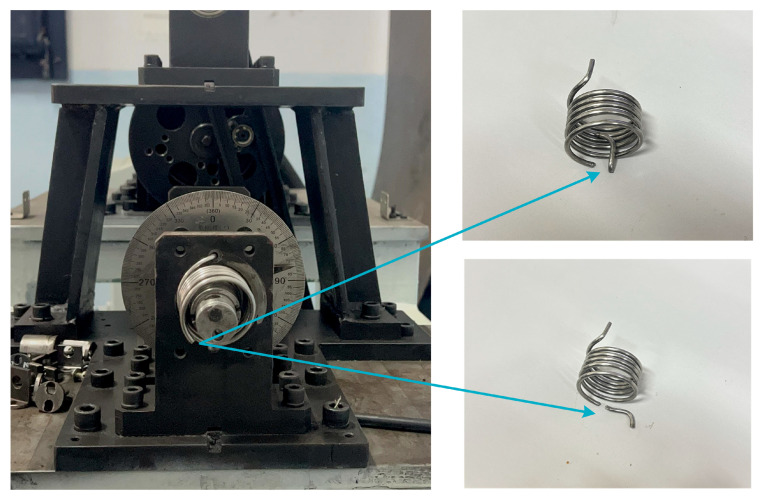
Experimental fracture torsion spring.

The spring was loaded cyclically under the set conditions until the damage variable according to the customized continuous damage mechanics reached 1, and the life of the spring was the number of cycles at the moment when the damage reached 1. It can be seen that the theoretically predicted fatigue fracture region was at the end of the spring at the torsion arm connection, which was also located in the inner ring of the spring (in contact with the fixed guide bar), where the fracture diagram is shown in Figure 11, which is in agreement with the actual fracture of the physical situation. The consistency of this result further verified the applicability of predicting the fatigue life of titanium torsion springs by fatigue theoretical modeling.

### 4.2. Analysis of Damage Variables

**Figure 12:**
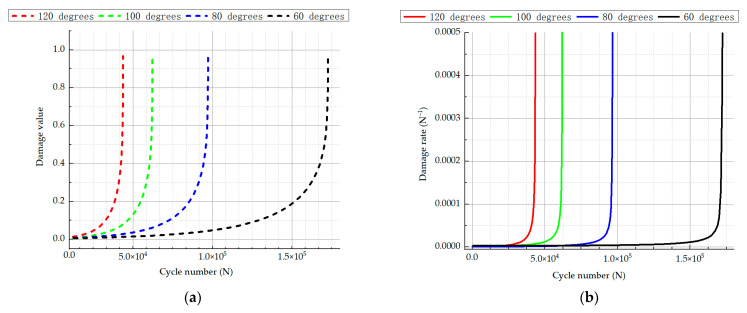
(**a**) Relationship between different torsion angles and damage values; (**b**) relationship between different torsion angles and damage rate.

A key advantage of fatigue damage analysis using continuous damage mechanics is its ability to accurately capture nonlinear damage evolution. This approach also provides a clear depiction of the local damage progression. The curve data corresponding to the number of cycles were extracted from the damage value at the danger point, as shown in Figure 12a. This figure shows that the damage evolution increased faster and faster with the increase in the number of cycles. During the early and middle stages of fatigue, the cumulative damage in the spring was relatively low. As the number of cycles increased, the damage at the critical point gradually built up and expanded. As fatigue failure approached, the damage at this critical point increased rapidly, where it reached a level of 1, indicating complete damage. It can be seen in Figure 12b that the trend diagram of the damage rate and the trend of damage value were roughly the same, showing a phenomenon that the growth rate was relatively slow in the early stage of the cycle and the fatigue limit of the spring itself increased sharply. It can be concluded that when the damage variable of the spring was close to 1, the damage degree of the spring accumulated rapidly and reached the break within a short cycle.

### 4.3. Comparison of Experimental and Theoretical Predictions

**Figure 13:**
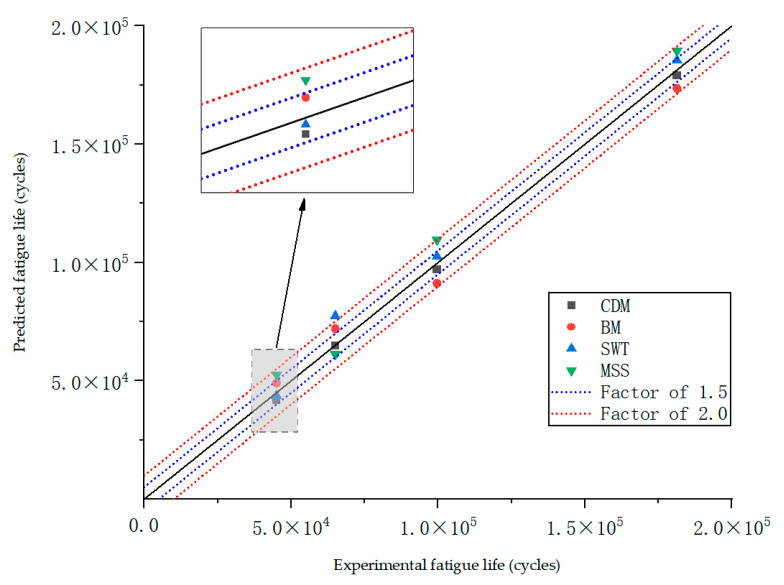
Comparison of test lives from different prediction models.

To assess the applicability and reliability of the UMAT subroutine based on continuum damage mechanics for the fatigue life prediction of titanium alloy torsion springs, the predicted life of this paper’s model (CDM), the MSS model [39,40], the SWT model [41,42], and the BM model [43,44] were compared with the experimental life. As shown in Figure 13, where the blue dotted line indicates the 1.5-fold error band, the red dotted line indicates the 2-fold error band, and the black solid line indicates that the predicted values were equal to the measured values. Figure 13 shows the results of the fatigue life comparison under different torsion angles, and it can be seen that the MSS criterion was close to 2 times the error under cyclic loading at 60 and 80 degrees, and the life prediction obtained by the SWT criterion at 100 degrees was beyond the range of 2 times of the experimental fatigue life such that the predicted results of the SWT criterion had the poorest correlation with the experimental results. For the BM criterion, the life prediction exceeded the experimental fatigue life by 1.5 times. For the criterion based on continuous damage mechanics, the predicted life under torsional cycling at different angles complied well with the 1:1.5 correlation, i.e., the predicted value was within 1.5 times the test fatigue life.

Through comparison, it was found that the fatigue life predicted by the continuous damage model was in good agreement with the life obtained by the actual experiment. It should be noted that in continuous damage mechanics, when the damage variable D attains a value of 1, it theoretically indicates that the spring material sustained damage, leading to the rapid propagation of cracks to a macroscopic size under cyclic loading. However, this does not imply that the specimen was instantly fractured. Consequently, the fatigue life predicted by this method was considered conservative. The relative error was used to evaluate the accuracy of model predictions by calculating the relative error between each predicted value and the experimental value:

(28) $$\mathrm{APE}=\frac{1}{n}\sum_{i=1}^{n}\left|\frac{y_{i}-{\hat{y}}_{i}}{y_{i}}\right|\times 100\%$$

where $y_{i}$ is the real value, ${\hat{y}}_{i}$ is the predicted value, and *n* is the number of experimental conditions.

**Figure 14:**
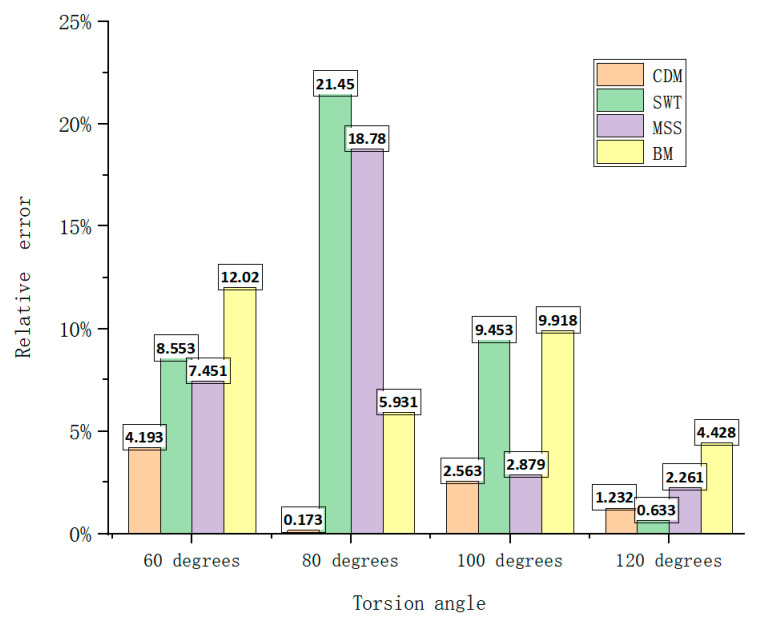
Relative errors of different prediction models for four torsion angles.

Figure 14 shows the absolute errors of the different prediction models for different torsion angles and Table 6 shows the average absolute errors. It can be seen that the average absolute error of the CDM model was the smallest under different cyclic loading conditions of the torsion spring, indicating that the CDM model had a relatively accurate prediction accuracy under the comprehensive loading conditions of the spring. Although the SWT model had a good prediction accuracy under 120-degree cyclic loading, the CDM model was the most accurate at predicting the fatigue life of the titanium alloy torsional spring when considering the total error under different cyclic loading conditions.

This result further proved that the prediction method based on continuous damage mechanics had good accuracy compared with other multi-axis prediction methods. In summary, the model presented in this paper demonstrated good predictive performance under cyclic loading with different torsional angles.

## 5. Conclusions

1. Based on the continuous damage mechanics model combined with the secondary development of the UMAT subroutine, a damage mechanics finite element numerical method for predicting the fatigue life of torsion springs of Ti-6Al-4V alloys was established and compared with other multi-axial fatigue prediction models, and the average relative error between the customized continuum damage mechanics subroutine and the actual measured spring life was 2.04%, which shows a good prediction accuracy as compared with other prediction models.
2. The finite element simulation based on the continuous damage fatigue model could represent the damage evolution process, which was of great significance for understanding the fatigue damage mechanism.
3. The fracture region of the spring predicted by the model agreed with the experimentally derived fracture region of the spring. Comparing the fatigue life results of the prediction model with the experimental results in different dimensions verified the validity and applicability of the proposed model and method, reduced the time and economic costs due to the long experimental cycle, and has certain reference value for improving the accuracy of fatigue life prediction of this type of titanium alloy torsion springs. The research results can provide an effective reference for the further optimization of the design of this titanium alloy torsion spring.
4. Suggestions for the future: this study did not consider the effect of surface area defects on the fatigue performance of springs, and the effects of various other surface treatments (shot peening and coatings) on the fatigue performance of springs may be investigated over time in order to improve their structural integrity and fatigue life. Suggestions for model enhancement: the parameters of the fatigue prediction model can be adjusted and optimized by combining machine learning and artificial intelligence algorithms with the experimental fatigue life to make the model predictions closer to the experimental fatigue life, which will potentially improve the accuracy of the prediction model.

## Figures and Tables

**Table 1:** Stresses corresponding to different mesh densities at 120-degree torsion angles.

Mesh Size/mm	Maximum Stress/MPa
1.5 mm	1108.7
1 mm	1108.9
0.5 mm	1109
0.2 mm	1109.2

**Table 2:** The fatigue life of the experimental spring without an initial torsion angle.

Angle of Torsion	60 Degrees	80 Degrees	100 Degrees	120 Degrees
Fatigue life	181,322	99,677	65,067	45,070

**Table 3:** Mechanical properties and material parameters of Ti-6Al-4V.

E (MPa)	v	$\sigma_{y}$ (MPa)	$C_{1}$ (MPa)	$C_{2}$ (MPa)	$r_{1}$	$r_{2}$
116,000	0.34	965	136,500	8100	1050	45

**Table 4:** Parameters of Ti-6Al-4V elastic damage evolution model.

$\sigma_{u}$ (MPa)	$\sigma_{l0}$ (MPa)	β	$aM_{0}^{-\beta }$	$b_{1}$	$b_{2}$	a
1180	358	2.1	1.79 × 10^−11^	0.0013	0.00055	0.75

**Table 5:** Parameters of Ti-6Al-4V plastic damage evolution model.

S	m
9.9293	4.7846

**Table 6:** Mean relative errors (MREs) of prediction models.

Model	Mean Relative Error (MRE)
CDM	2.04%
SWT	10%
MSS	7.84%
BM	8%

## Data Availability

The original contributions presented in this study are included in the article. Further inquiries can be directed to the corresponding author.
